# Circulating Tumor and Invasive Cell Gene Expression Profile Predicts Treatment Response and Survival in Pancreatic Adenocarcinoma

**DOI:** 10.3390/cancers10120467

**Published:** 2018-11-24

**Authors:** Kenneth H. Yu, Mark Ricigliano, Brian McCarthy, Joanne F. Chou, Marinela Capanu, Brandon Cooper, Andrew Bartlett, Christina Covington, Maeve A. Lowery, Eileen M. O’Reilly

**Affiliations:** 1 Memorial Sloan Kettering Cancer Center, New York, NY 10065, USA; chouj@mskcc.org (J.F.C.); capanum@mskcc.org (M.C.); christinamonique92@gmail.com (C.C.); mlowery@tcd.ie (M.A.L.); oreillye@mskcc.org (E.M.O.); 2 Weill Cornell Medical College, New York, NY 10065, USA; 3 Adera Biolabs, Germantown, MD 20876, USA; mark.ricigliano@aderabio.com (M.R.); brian.mccarthy@aderabio.com (B.M.); bcooper@som.umaryland.edu (B.C.); andrew.bartlett@aderabio.com (A.B.)

**Keywords:** circulating tumor and invasive cells, pancreatic cancer, *SMAD4*, gemcitabine, nab-paclitaxel, 5-fluorouracil, FOLFIRINOX

## Abstract

Previous studies have shown that pharmacogenomic modeling of circulating tumor and invasive cells (CTICs) can predict response of pancreatic ductal adenocarcinoma (PDAC) to combination chemotherapy, predominantly 5-fluorouracil-based. We hypothesized that a similar approach could be developed to predict treatment response to standard frontline gemcitabine with nab-paclitaxel (G/nab-P) chemotherapy. Gene expression profiles for responsiveness to G/nab-P were determined in cell lines and a test set of patient samples. A prospective clinical trial was conducted, enrolling 37 patients with advanced PDAC who received G/nab-P. Peripheral blood was collected prior to treatment, after two months of treatment, and at progression. The CTICs were isolated based on a phenotype of collagen invasion. The RNA was isolated, cDNA synthesized, and qPCR gene expression analyzed. Patients were most closely matched to one of three chemotherapy response templates. Circulating tumor and invasive cells’ *SMAD4* expression was measured serially. The CTICs were reliably isolated and profiled from peripheral blood prior to and during chemotherapy treatment. Individual patients could be matched to distinct response templates predicting differential responses to G/nab-P treatment. Progression free survival was significantly correlated to response prediction and Δ*SMAD4* was significantly associated with disease progression. These findings support phenotypic profiling and Δ*SMAD4* of CTICs as promising clinical tools for choosing effective therapy in advanced PDAC, and for anticipating disease progression.

## 1. Introduction

Pancreatic ductal adenocarcinoma (PDAC) currently represents the 3rd leading cause of cancer mortality in the U.S. Of the five most lethal cancers, incidence and death rates are only increasing for PDAC. Therefore, it is estimated that by 2020, PDAC is likely to rise to the 2nd leading cause of cancer death in the U.S. [1]. Despite this, the emergence of active combination chemotherapy regimens during the past three years has led to incremental improvements in overall survival. Combination chemotherapy regimens, such as 5-fluorouracil (5-FU), leucovorin, irinotecan, oxaliplatin (FOLFIRINOX) [2], and gemcitabine with nab-paclitaxel (G/nab-P) [3] represent clinically meaningful improvements over the prior standard of care, single agent gemcitabine, for patients with advanced PDAC.

Clinicians remain without tools for predicting which of these chemotherapeutic agents will be most effective for treating individual patients with PDAC. In other diseases, tumor tissue-based biomarkers have been identified as predictive of drug effect. Typically, this strategy has been effective where the biomarker is linked to the mechanism of action of the agent, i.e., Her2 expression and trastuzumab or activating EGFR mutation and erlotinib. Similar strategies have generally not been successful in PDAC, largely due to the absence of active, targeted therapies. A recent example attempting to predict response to cytotoxic chemotherapy involved the human equilibrative nucleoside transporter-1 (hENT1), a transporter protein thought important for cellular uptake of gemcitabine. Preliminary studies suggested low expression of hENT1 could result in gemcitabine resistance; however, prospective validation did not confirm these findings in patients with advanced disease [4].

An alternative approach is based on the connectivity map concept [5]. Connectivity mapping hypothesizes that biological systems with similar gene expression profiles share biological properties important for drug response. The connectivity mapping approach has been validated, for example, effectively predicting rapamycin induced glucocorticoid sensitivity in acute lymphoblastic leukemia [6]. We hypothesize that gene expression profiles of response to anticancer agents can be created by comparing the expression patterns of model systems, such as cell lines, with divergent drug responses. Tumors with gene expression profiles similar to drug resistant profiles will be resistant to treatment, and those similar to drug sensitive profiles will be responsive.

Translating a tumor-based drug prediction model to a blood-based assay is attractive and feasible. A clinical assay based on circulating cells found in peripheral blood has several advantages over one based on tumor tissue. Peripheral blood can be sampled repeatedly over time conveniently and safely, unlike tumor tissue. Circulating cells found in peripheral blood may provide unique information regarding cancer growth and metastasis not present in any individual tumor. We have built a robust platform for capturing and preserving circulating cells from 6 mL of heparinized blood drawn peripherally from patients with PDAC. This approach has been previously shown to successfully isolate circulating tumor cells (CTCs) with tumorigenic properties (CD45-EpCAM+CK+ and the ability to degrade and ingest collagenous matrices) in breast [7], prostate [8], and ovarian [9] cancers. Circulating tumor cells isolated in this fashion have been shown to correlate numerically with cancer stage [7] and prognosis [9]. Furthermore, these CTCs have been shown to reflect the genomics of the primary tumor [8], including presence of *KRAS* mutation in CTCs isolated from PDAC patients [10]. Not all captured cells express these markers typical of classical tumor cells, but all cells isolated in this manner have the ability to invade into cell-adhesion matrices. In a prior study, CTCs made up between 0.03 to 0.07% of the cells isolated [10]. This modified cell invasion assay isolates classical CTCs [11] and invasive immune cells (EPCAM(−) mesenchymal cells, and invasive immune cells), hence the term circulating tumorigenic and invasive cells (CTICs) [12,13,14].

In a previous study using a different drug sensitivity approach, gene expression profiling of CTICs was shown to predict effective therapy in advanced PDAC [15]. This study was conducted in a cohort of patients (*n* = 50) treated predominantly with 5-FU based chemotherapy, prior to the FDA approval of G/nab-P, which has now become a widely used standard frontline treatment for advanced PDAC.

Beyond predicting effective therapy, clinicians are also without tools or biomarkers that can anticipate treatment response or resistance in individual patients with PDAC. The most commonly used circulating biomarker for monitoring PDAC is the Sialyl Lewis A antigen, carbohydrate antigen (CA) 19-9. CA 19-9 has limited prognostic power in PDAC. Low and decreasing CA 19-9 levels following surgical resection [16,17] have been shown to correlate with survival. In patients with locally advanced PDAC, a decrease of >90% in CA 19-9 level following chemoradiotherapy has been shown in one study to be associated with improved survival [18]. In patients with advanced PDAC, however, CA 19-9 is a poor predictor of survival and results show heterogeneous outcomes. One large randomized study found that a 50% decrease in CA 19-9 after two months of chemotherapy treatment or at CA 19-9 nadir did not predict for longer survival in advanced PDAC [19]. Furthermore, CA 19-9 serum level is limited by poor sensitivity, false negative results in Lewis negative phenotype (5–10%), and increased false positivity in the presence of obstructive jaundice (10–60%) [20].

*SMAD4* is one of the four most commonly mutated genes seen in PDAC, along with *KRAS*, *TP53* and *CDKN2A*. Mutational rate seen in a recent large sequencing effort was 35% [21]. *SMAD4* is a classical tumor suppressor gene, and acts to regulate the transforming growth factor-β (TGF-β) signaling pathway [22]. Evidence is accumulating associating *SMAD4* loss with poor prognosis in PDAC, therefore, *SMAD4* expression is a biomarker of great interest in PDAC.

The current study demonstrates that, using an innovative phenotypic PGx model applied to CTICs isolated from a single blood sample, patients with advanced PDAC can be divided into three treatment response groups. This profile is predictive of progression-free survival, and a trend for overall survival. Serial measurement of *SMAD4* expression is also predictive of treatment response and resistance.

## 2. Results

### 2.1. Rate of Successful CTIC Gene Expression Profiling

From a single 6 mL heparinized whole blood sample, CTICs could be isolated, greater than 1.0 ng/μL of RNA extracted and PGx profiling successfully performed in >95% of the samples analyzed. Analysis failure could be attributed to errors in qPCR, typically isolated to failure of a single gene to amplify, bad passive dye readings and exponential algorithm failures. Errors in EPCAM+ cell isolation were attributed to sample degradation during shipment.

### 2.2. Phamacogenomic (PGx) Profiles

A PGx model was developed (see Materials and Methods). A prospective clinical trial was conducted to validate this model. All 37 patients enrolled in the study received frontline treatment with G/nab-P. Seven patients were excluded from analysis due to early discontinuation of treatment prior to restaging, either due to treatment toxicity (*n* = 3) or death (*n* = 4). All PDAC patient blood samples drawn prior to frontline treatment could be matched to one of three distinct drug sensitivity profiles: G/nab-P, intermediate or FOLFIRINOX. A strong negative correlation was seen between profiles predicting sensitivity to G/nab-P and FOLFIRINOX (see Figure S1, R^2^ = 0.74, *p* = 0.0001). At baseline, 40% (12/30) of evaluable patients were predicted to be most sensitive to G/nab-P, 23% (7/30) of intermediate sensitivity or 37% (11/33) most sensitive to FOLFIRINOX (see Table 1 for patient demographics). With respect to the G/nab-P regimen specifically, 57% (17/30) patients were predicted to have sensitivity, 43% (13/30) resistance. Patient characteristics were balanced across phenotypic profiles. Patient age was significantly positively associated with both progression free (PFS) and overall survival (OS), however, no association was seen between age and PGx prediction.

### 2.3. PGx Profile Predicts PFS

**Figure 1:**
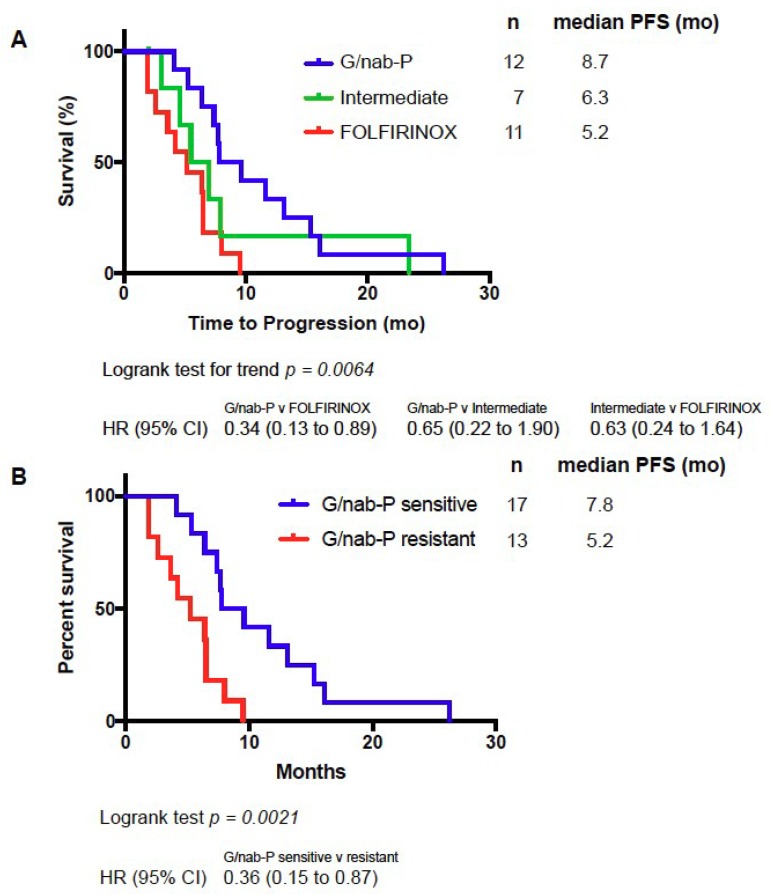
Kaplan–Meier analysis of progression-free survival (PFS) of patients treated with G/nab-P based on classification into one of three PGx profiles (**A**), and based on predicted G/nab-P sensitivity (**B**).

**Figure 2:**
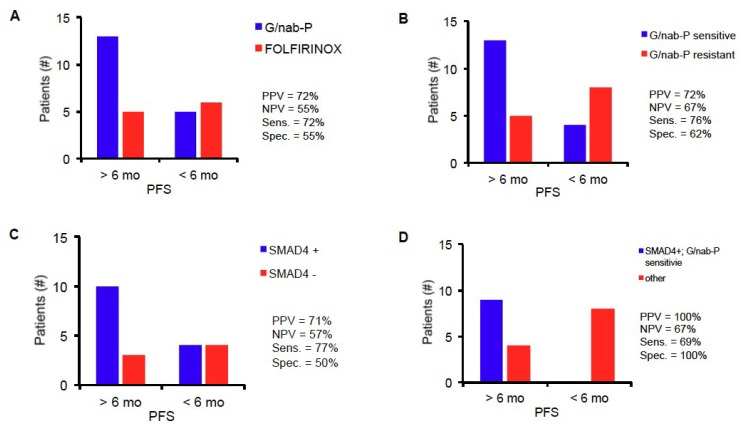
Performance of PGx profiling (**A**), G/nab-P sensitivity (**B**), Δ*SMAD4* (**C**), and a combined analysis (**D**) to predict treatment response. Six-month PFS was used as a cut-off for predicting sensitivity and response (>6 months) or resistance and lack of response (<6 months).

Median PFS for all evaluable patients in this study was 7 months. We hypothesized that patients with profiles predicting drug sensitivity to G/nab-P would experience the longest PFS; patients with profiles predicting drug sensitivity to FOLFIRINOX, and therefore resistance to G/nab-P based on our preclinical modeling, would experience the shortest PFS; and patients with profiles predicting intermediate drug sensitivity would experience an intermediate PFS. Significant differences in PFS were in fact seen in patients from the three PGx profile groups. Median PFS for the PGx profile groups G/nab-P, intermediate and FOLFIRINOX groups were 8.7, 6.3, and 5.2 months, respectively (see Figure 1A). Comparing PFS across the three groups, log-rank test for trend was statistically significant, *p* = 0.0064. Comparing the G/nab-P to FOLFIRINOX groups only, PFS was significantly longer in the G/nab-P group (*p* = 0.0031, hazard ratio of 0.34, log-rank test). Six-month PFS (PFS-6) is a commonly used surrogate endpoint for assessing drug response [23,24]. Using PFS-6 as a cutoff, PGx profile grouping had a positive predictive value (PPV) of 72%, negative predictive value (NPV) of 55%, sensitivity of 72%, and specificity of 55% (see Figure 2A).

Analysis was performed based on G/nab-P sensitivity or resistance. Patients with predicted G/nab-P sensitivity demonstrated a median PFS of 7.8 vs. 5.2 months for those with predicted resistance (*p* = 0.0021, see Figure 1B). Using 6-month PFS as a cutoff, G/nab-P drug sensitivity prediction has a PPV of 72%, NPV of 67%, with a sensitivity of 76% and specificity of 62% (see Figure 2B).

Median overall survival (OS) for all evaluable patients in this study was 12.5 months. A trend for OS difference was seen based on G/nab-P sensitivity or resistance (median OS 12.6 vs. 9.8 months, *p* = 0.07). Of note, the vast majority of patients who went on to second line therapy (17/18, 94%) received 5-fluorouracil based chemotherapy.

### 2.4. Change in SMAD4 Level Predicts PFS and Response to Treatment

**Figure 3:**
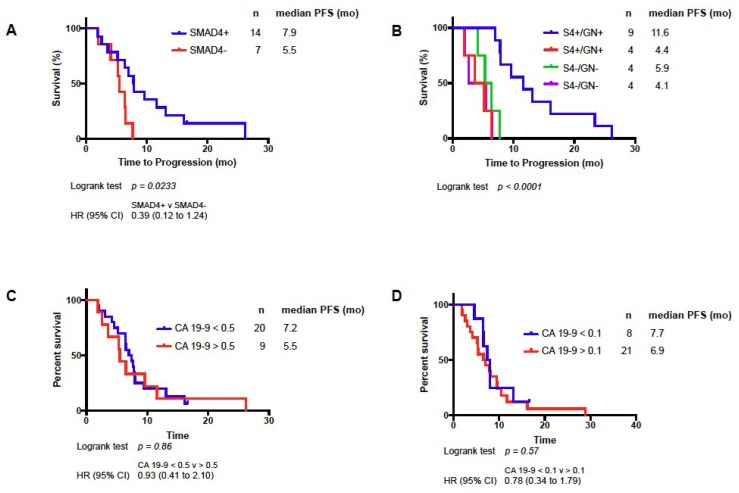
Kaplan–Meier analysis of PFS based on Δ*SMAD4* (**A**), Δ*SMAD4* (S4), and G/nab-P sensitivity (GN+) or resistance (GN-); (**B**) decrease in carbohydrate antigen (CA) 19-9 by 50% (**C**) or 90% (**D**) after 2 months of treatment with G/nab-P.

Longitudinal *SMAD4* measurements were obtained for 21 patients. Measurements were made from CTICs obtained prior to treatment and after 8–12 weeks of treatment. Increase in *SMAD4* expression, termed *SMAD4*+, was seen in 14 patients, and decrease expression, termed *SMAD4*-, was seen in seven patients. *SMAD4*+ patients experienced significantly longer PFS compared to *SMAD4*- patients (7.9 mo versus 5.5 mo, HR = 0.39, log-rank test, *p* = 0.0233, Figure 3A). Using 6-month PFS as a cutoff, *SMAD4*+ had a PPV of 71%, NPV 57%, with a sensitivity of 77% and specificity of 50% (see Figure 2C). Baseline level of *SMAD4* expression was not predictive of PFS, and tumor tissue *SMAD4* expression was not available. Historically, change in CA 19-9 was used as a biomarker of disease response to chemotherapy. Baseline and change in CA 19-9 from baseline to after 8–12 weeks of treatment were analyzed. Baseline CA 19-9 was not predictive of PFS. Using thresholds of 50% and 90% CA 19-9 decrease, no PFS difference was seen (Figure 3C,D). Combining Δ*SMAD4* and CA 19-9 was not predictive of PFS.

Combining Δ*SMAD4* and PGx platforms, patients with profiles predicting response to G/nab-P and *SMAD4*+ (S4+/GN+) experienced impressive PFS of 11.6 months, much longer than any of the other subgroups in the analysis (between 4.1 and 5.9 months, *p* < 0.0001, see Figure 3B). Using 6-month PFS as a cutoff, this combined profile (S4+/GN+) has a PPV of 100%, NPV of 67%, with a sensitivity of 79% and specificity of 100% (see Figure 2D).

## 3. Discussion

Advanced PDAC remains incurable; however, the development of combination chemotherapy regimens such as G/nab-P [3] and FOLFIRINOX [2] has resulted in more effective treatment options. Clinicians remain without effective tools for choosing the most active treatment regimen and for anticipating disease progression for individual patients. Researchers also require new and effective tools for screening new drugs. The current study supports PGx profiling of CTICs as a promising new tool for addressing these unmet needs.

The CTICs represent a mixed population of cells including classical EPCAM(+) CTCs, EPCAM(−) mesenchymal cells, and invasive immune cells. While isolation of pure, circulating tumor cells for study is worthwhile, such cells are present at exceedingly low levels, particularly in patients with PDAC. Nevertheless, a growing body of literature supports the utility of a mixed, CTIC population, particularly for predicting drug therapy in patients. For example, Pearl and colleagues, using a similar invasion assay in ovarian cancer, recently demonstrated that direct chemotherapy treatment of patient-derived CTICs in vitro accurately predicted in vivo response [11]. Our current study supports the intriguing concept that invasive cells present in circulation, the majority of which do not express markers consistent with classical CTCs, can be used to predict treatment response. CTICs merit further study to better understand the composition of this cell population and their biology. In PDAC, gene expression profiling rather than direct drug treatment of CTICs has been shown to be a reasonable surrogate for predicting drug response in vivo [14].

One approach to predicting drug response in PDAC is to focus only on PDAC derived models [25]. Development of the oncogenic *KRAS* genetically engineered mouse (GEM) model of PDAC has accelerated the screening of drug candidates for clinical testing [26]. It has become apparent, however, that tissue of origin is not sufficient for predicting drug responses in patients, with drugs targeting smoothened (SMO) and heparin sulfate showing promise in GEM models, but ultimately failing in the clinic. New, innovative patient-derived organoid (PDO) models may offer a way forward, capturing the complexity of the human disease [27]. Our recent work shows that drug responses in PDOs parallel the response of patients from whom they are derived. Gene expression profiles of PDOs can be generated which also can predict response to some drugs in patients.

Our current study is based on the premise that gene expression pathways relevant to drug response are independent of, and more predictive than, tissue of origin. This hypothesis builds upon prior work using the NCI-60 cell line panel to model drug sensitivity based on common genes and pathways across tumor types. Scherf and colleagues previously demonstrated that a simple cluster analysis could segregate the NCI-60 cell lines primarily based on their tissue of origin; however, when cell lines were clustered based on growth inhibition of 1400 drugs, clustering differed greatly, based on gene expression profiles [28]. Cell lines with common tissues of origin often demonstrated disparate drug responses. Strong mechanistic rationale was found for the genes predictive of drug response, for example, dihydropyrimidine dehydrogenase expression and 5-FU, and asparagine synthetase (ASNS) expression and L-asparaginase. Staunton and colleagues [29] further explored this approach, using the NCI-60 cell lines to study response to 232 drugs. A training group of cell lines were used to generate profiles of sensitivity and resistance. The profiles were then tested in a separate validation group. Profiles were designed independent of tissue of origin. Eighty-eight of the 232 profiles accurately predicted drug sensitivity. Lee and colleagues [30] further extended this approach, using the NCI-60 panel to generate a novel gene expression algorithm termed coexpression extrapolation, or COXEN, to predict drug sensitivity. The algorithm was accurate for predicting drug response in breast cancer patients, and cisplatin response in an independent panel of 40 bladder cancer cell lines. Importantly, bladder cancer, like pancreatic cancer, is not a tumor type included in the NCI-60 panel.

The current study demonstrates the effectiveness of a platform which is innovative and distinct from this previously developed and published platform. In the prior study, drug sensitivity profiles were developed and matched to patient CTIC gene expression profiles using gene-set enrichment analysis. The current study uses an innovative nearest template prediction approach (Adera Biolabs, Germantown, MD, USA). The previously described assay was based on a standard microarray platform, whereas the current study utilizes qPCR, which is regarded as the gold standard for measuring RNA expression [31,32]. The microarray approach has several advantages such as the ability to measure expression of thousands of genes simultaneously and reasonable accuracy. Disadvantages include high background signal due to cross-hybridization and limited dynamic range of detection. At the time that the expression profiles were generated from the NCI-60 panel, currently available next-generation RNA sequencing (RNAseq) was not available. RNAseq allows for more reliable quantitation of large numbers of transcripts, compared with microarray approaches, and is the basis of our current PDO profiling work. The current PGx assay profiles expression of more than 80 genes not assessed in previous studies (propriety to Adera BioLabs, Germantown, MD); the primary role of these genes is to transport drugs and other molecules. Several of the genes in the current profile have been characterized, typically in cell lines or tumor tissue, and have been found to play an important role in response and resistance to cytotoxic chemotherapeutic drugs. Our current study includes assessment of select *ABC* superfamily gene members (including *ABCB1*, *ABCC1*, *ABCC2* and *ABCG1*) known to act as active drug transporters to reduce accumulation of chemotherapy drugs within resistant cancer cells [33,34,35,36]. More recently, solute carrier (SLC) genes have been studies as important genes governing drug sensitivity and resistance. In total, 400 SLC genes have been identified, acting to transport a variety of molecules across the plasma membrane or in intracellular organelles [36,37]. Germline polymorphisms in both *ABC* and *SLC* genes have been linked to chemotherapy resistance in colon cancer [38]. Expression of *SLC28A1* and *SLC29A1* in PDAC tumor tissue have been best studied and are associated with gemcitabine response [39]. Other members of the SLC family have been studied to a lesser extent and association with drug response is largely unknown.

Our results represent the first set of studies demonstrating that expression profiling of a panel of these genes in CTICs can predict treatment response. In patients with advanced PDAC, our PGx model identifies three groups with differential response to cytotoxic chemotherapy regimens. In an independent group of patients with advanced PDAC, all of whom were treated with G/nab-P chemotherapy, patients predicted to respond to G/nab-P experienced significantly longer PFS compared to patients predicted not to respond to G/nab-P. While there is a trend to longer OS in the G/nab-P sensitive group, this does not reach statistical significance; possible explanations include the small size of the study, and the high percentage of patients who received 5-FU based chemotherapy in the second line. For patients predicted to be resistant to G/nab-P, sensitivity to 5-FU based chemotherapy could account for similar OS. A prospective study, using our assay to direct chemotherapy treatment in multiple lines of therapy, is needed to confirm PFS and OS benefits, and is underway.

Profiling and drug response prediction can be performed on a single 6 mL blood sample. The current study, focused on patients receiving G/nab-P chemotherapy, a recently developed front-line standard therapy for advanced PDAC, together with results of a prior study, which enrolled patients receiving a variety of different chemotherapy regimens, primarily built upon a 5-FU backbone [15], supports the concept of PGx profiling of CTICs as a clinically useful assay to help clinicians choose effective chemotherapeutic regimens for patients with advanced PDAC.

*SMAD4* is a biomarker of great interest and has been studied extensively in PDAC tissue. One early study described patterns of metastases at time of autopsy in 76 patients with PDAC and that loss of *SMAD4* was highly correlated with the presence of a widespread metastasis [40]. A recent meta-analysis of 1762 patients from 14 studies concluded that loss of *SMAD4* expression in PDAC tumors was associated with poor overall survival [41]. Intriguingly, a genetically engineered mouse model suggests loss of one *SMAD4* allele leads to local disease progression but not distant metastases, and further loss of *SMAD4* heterozygosity leads to increase of both local and metastatic potential [42]. *SMAD4* expression has also been shown to be important for anti-tumor activity in circulating immune cells such as T cells [43] and NK cells [44]. Further mechanistic studies are warranted. The current study is the first evidence that diminished expression of *SMAD4* in CTICs also is a predictor of poor prognosis and outperforms CA 19-9 as a predictor of treatment response and resistance. With the emergence of new biomarker candidates, including a variety of nucleic acids (circulating DNA, lncRNA and miRNA [45], exosomes and proteins (thrombospondin-2 [46]), investigation into their role in predicting treatment response is warranted.

Prospective validation of both PGx profiling and *SMAD4* profiling in CTICs is currently underway (ClinicalTrials.gov Identifier NCT03033927). We envision CTIC profiling will emerge as a clinical useful tool for guiding standard combinations of cytotoxic chemotherapy in the near term. The workflow and assay are also being studied as a method for identifying patients who will be responsive to cytotoxic chemotherapy drugs not typically used for the treatment of PDAC, and to accelerate the development of new, experimental agents.

## 4. Materials and Methods

### 4.1. Preclinical Studies and Model Development

Five chemotherapeutic agents chosen for modeling were gemcitabine (G), nab-paclitaxel (nab-P), 5-FU, oxaliplatin, and irinotecan. Gene expression templates were created for each of these 5 chemotherapeutic agents using cell line expression patterns of five NCI-60 cell lines, each line chosen based on sensitivity to one of these five drugs. Gene expression data for the entire NCI-60 panel was generated using the Human Genome U133 Plus 2.0 Array (Affymetrix, Santa Clara, CA, USA) and are publicly available (NCBI Gene Expression Omnibus). From this set of over 40,000 transcripts, the current model uses greater than 80 drug transport genes as classifiers in the algorithm (see Table S1 for gene families; specific genes within each family are proprietary to Adera Biolabs, Germantown MD). Drug transport genes were of particular interest based on prior pharmacogenomic studies; however, the current templates were created independently of the gene expression profiles described in earlier studies [15].

### 4.2. Nearest Template Prediction

To match patient blood samples to cell line derived drug sensitivity templates, Adera Biolabs utilized nearest template prediction analysis (previously described in References [47,48,49]) with its underlying premise that if two samples share similar expression profiles of key, relevant genes, they will have similar drug responses. An independent set of 137 blood samples collected from patients with advanced PDAC prior to chemotherapy treatment was analyzed by Adera Labs, LLC for model development. For drug response, pharmacogenomic model development, a subset of 57 blood samples were studied. The CTICs were isolated from blood samples as described below. EPCAM expression in the CTICs isolated was performed by qPCR, mean Ct value = 27.3, (CV 6.6%, 95% CI 27.7–26.8). Nearest template prediction methodology was used to match chemotherapeutic templates to the PDAC sample and sort the templates in ranked-order to obtain sensitivity values for each treatment option (Adera Biolabs, Germantown MD). The analysis resulted in a rank order list of chemotherapy templates that were subsequently combined into three chemotherapy regimen templates: G/nab-P, FOLFIRINOX, and an intermediate template (termed “intermediate”) with sensitivity to both G/nab-P and 5-fluorouracil. G/nab-P [3] and FOLFIRINOX [2] are considered standard of care, frontline regimens. All 57 patient samples could be best matched to only one of these three chemotherapy regimen templates.

Baseline *SMAD4* expression level was established by isolating CTICs as described below from all 137 blood samples in the independent set, derived from advanced PDAC patients prior to treatment. *SMAD4* expression was measured by qPCR and defined as (40-*SMAD4*-Ct)/*GAPDH*-Ct). An average expression of 0.83 was found (range, 1.26–0.25).

### 4.3. Clinical Trial Design

**Figure 4:**
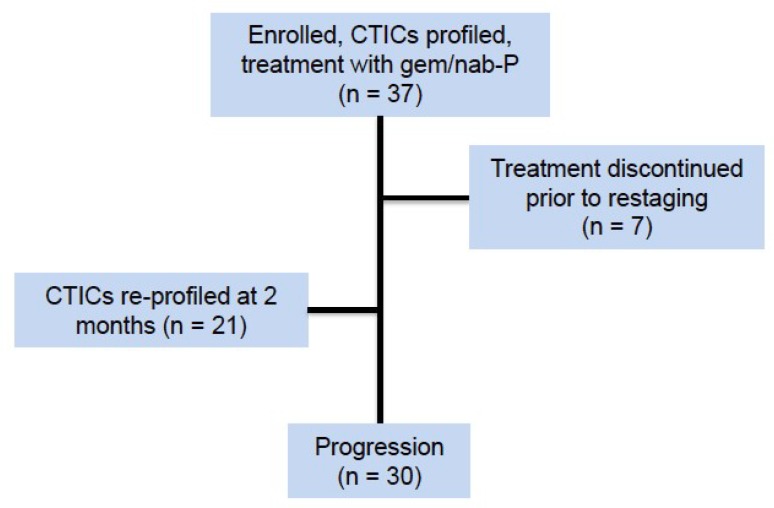
Consort diagram.

To validate the PGx model, a prospective observational non-randomized study was conducted at Memorial Sloan Kettering Cancer Center (MSKCC, registered at Clinicaltrials.gov, identifier NCT01474564 on 15 November 2011). All subjects gave their informed consent for inclusion before they participated in the study. The study was conducted in accordance with the Declaration of Helsinki, and the protocol was fully reviewed and approved by the MSKCC Institutional Review Board (IRB) and its Ethics Committee. The study was originally approved 12 October 2011, with an amendment covering the current study designed approved 04 December 2013. All research was performed in accordance with MSKCC institutional guidelines/regulations, and informed consent was obtained from all participants and/or their legal guardians. Enrollment occurred from January 2014 to January 2016. The primary objective of the study was (1) to assess the feasibility of obtaining and characterizing CTICs, (2) to use the resulting gene expression analysis to generate a treatment profile for patients with PDAC receiving gemcitabine and nab-paclitaxel, and (3) to assess candidate biomarkers of treatment response and progression. A total of 37 patients were enrolled, target enrollment was between 30 and 60 (see Table 1 for patient demographics). At the time of the current analysis, data cutoff was dated 15 March 2018, 30 patients were evaluable for treatment response, having met criteria for progression of disease or death (see Figure 4 for consort diagram). Key eligibility criteria included: histological or cytological confirmation of PDAC, radiographic confirmation of American Joint Committee on Cancer (AJCC) stage III or IV disease, planned treatment with G/nab-P and an Eastern Cooperative Oncology Group (ECOG) performance status of 0, 1 or 2.

Following written informed consent and prior to initiation of chemotherapy treatment, a 6-mL blood sample was obtained in a sodium-heparinized Vacutainer tube (Becton Dickinson, Franklin Lakes, NJ, USA) from each study participant using standard clinical procedures. Blood samples were collected by venipuncture or by accessing an indwelling catheter normally used for phlebotomy. Additionally, blood samples were collected and processed as described at first restaging, following 8–12 weeks of therapy, and at the time of disease progression.

### 4.4. Cell Enrichment

Coded and deidentified samples were shipped at 4 °C overnight to Adera Biolabs (Germantown, MD, USA) for CTIC isolation and enrichment. A collagen adhesion matrix (CAM) in a modified cell invasion assay was used to capture EPCAM+ invasive cells, a well-characterized approach for capturing CTCs [7,8,9,15]. 2.0 mL aliquots of whole blood were incubated with collagen-coated microcarriers (Pall Corporation, Port Washington, NY, USA) and cultured for 2 h in Dulbecco’s modified Eagle’s medium with F12 supplemented with 10% calf serum, 5% Nu-serum, 1 unit/mL penicillin, and 10 μg/mL streptomycin). Captured cells were then washed and lysed in situ.

### 4.5. qPCR and Expression Analysis

Lysed CTICs directly isolated from the invasion assay were subjected to qPCR analysis. Specifically, total RNA from lysed cells was purified by RNeasy Mini Kit (Qiagen, Valencia, CA, USA), cDNA was synthesized (Ovation Pico SL, Nugen Technologies, San Carlos, CA, USA) and then subjected to qPCR analysis. Gene expression of our 80+ gene panel was measured by qPCR and processed at standard thermal cycling rates using standard SYBR Green and ROX Mastermix (Adera Biolabs, Germantown, MD, USA). Arrays with >5% error rates were discarded.

### 4.6. *SMAD4* Analysis

CTIC *SMAD4* expression level was measured at baseline and at time of radiographic restaging, typically 8 to 12 weeks after starting therapy. Patients were classified as *SMAD4*+ if expression increased at time of restaging, or *SMAD4*- if expression decreased. Matched samples were obtained from 21 patients at baseline and at 1st restaging. Independently, gene expression levels of *ABCG2* and *ALDH1A1* were investigated as biomarkers of disease progression. Changes in *ABCG2* and *ALDH1* expression were not statistically significant between baseline and 1st restaging or baseline and disease progression.

### 4.7. Data Availability Statement Format Guidelines

The data that support the findings of this study are available from Adera Biolabs, Germantown MD, but restrictions apply to the availability of these data, which were used under license for the current study, and so are not publicly available. Data are however available from the authors upon reasonable request and with permission of Adera Biolabs, Germantown MD.

## 5. Conclusions

Although PDAC remains an intractable disease, effective chemotherapeutic regimens have recently been developed. We remain without effective tools for matching individual patients to effective treatments. The current clinical trial validates an innovative, pharmacogenomic assay of peripheral blood circulating tumors and invasive cells for predicting response and progression to the standard chemotherapy regimen of gemcitabine and nab-paclitaxel. This study provides confirmation that an individualized approach to treating pancreatic cancer is feasible, and testing against a wider range of chemotherapeutic agents is currently underway.

## Figures and Tables

**Table 1:** Patient demographics.

	All Study Participants	Non-Evaluable	Evaluable	Gem/nab-P	Treatment Profile	FOLFIRINOX	Δ*SMAD4*	
					Intermediate		Increase (+)	Decrease (−)
No. of patients (%)	37	7	30	12 (40)	7 (23)	11 (37)	14	7
Mean age	71.9	73.3	71.5	72.8	73.8	68.7	73.4	70.6
Gender								
male	21	6	15	5	3	7	6	4
female	16	1	15	7	4	4	8	3
Stage								
III	2	1	1	0	0	1	0	0
IV	35	6	29	12	7	10	14	7
Performance status								
ECOG 0	3	0	3	1	0	2	2	0
ECOG 1	24	3	21	7	7	7	7	6
ECOG 2	10	4	6	4	0	2	5	1

Eastern Cooperative Oncology Group (ECOG), 5-fluorouracil (5-FU), leucovorin, irinotecan, oxaliplatin (FOLFIRINOX), gemcitabine with nab-paclitaxel (G/nab-P).

## Supplementary Materials

The following are available online at http://www.mdpi.com/2072-6694/10/12/467/s1. Figure S1: Correlation of PGx score comparing FOLFIRINOX and G/nab-P pairwise for each study participant, Table S1: Gene families used for PGx model.
