# Supplementary material for: Circulating Tumor and Invasive Cell Gene Expression Profile Predicts Treatment Response and Survival in Pancreatic Adenocarcinoma
Source: Cancers (Basel). 2018 Nov 24;10(12):467. doi: 10.3390/cancers10120467 (PMC6315371; doi:10.3390/cancers10120467)
Supplement: Supplementary file 1 [file cancers-10-00467-s001.pdf]

Supplementary Materials: Circulating Tumor and Invasive Cell Gene Expression Profile Predicts Treatment Response and Survival in Pancreatic Adenocarcinoma

Kenneth H. Yu, Mark Ricigliano, Brian McCarthy, Joanne F. Chou, Marinela Capanu, Brandon Cooper, Andrew Bartlett, Christina Covington, Maeve A. Lowery and Eileen M. O'Reilly

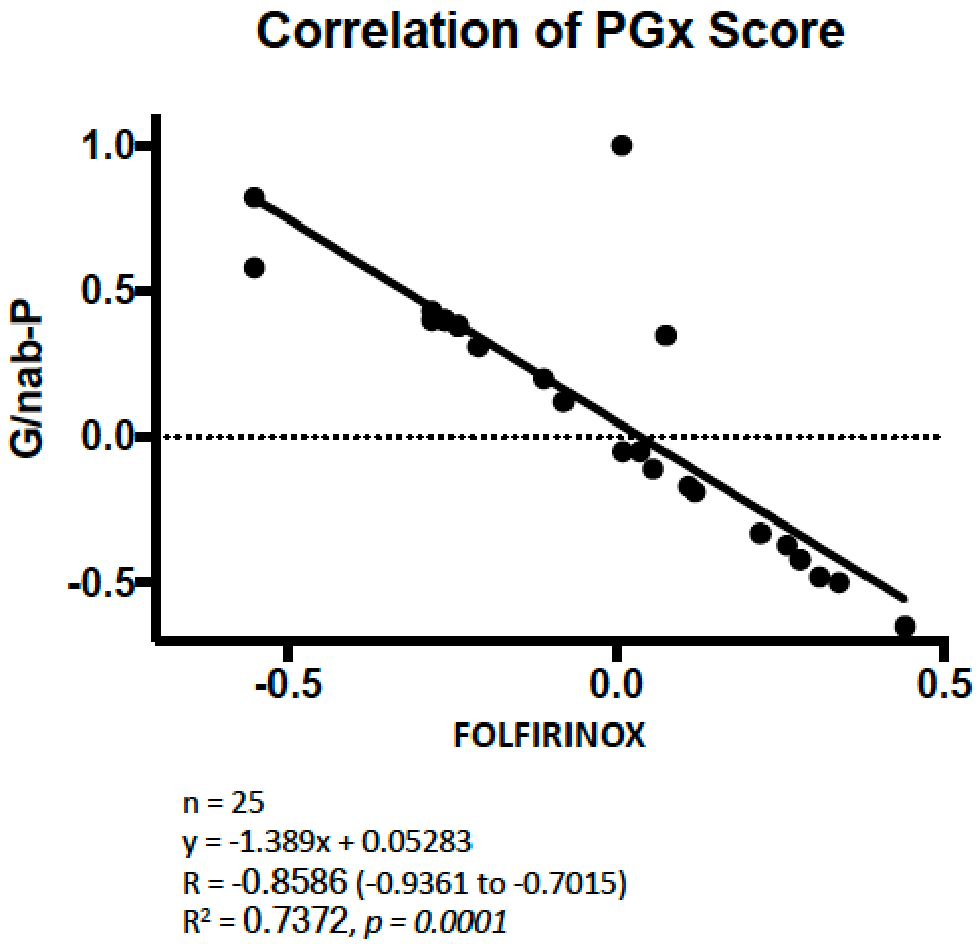

Figure S1. Correlation of PGx score comparing FOLFIRINOX and G/nab-P pairwise for each study participant.

Table S1. Gene families used for PGx model.

ABCA
ABCB
ABCC
ABCD
ABCF
ABCG
AQP
MVP
SLC2
SLC3
SLC5
SLC7
SLC10
SLC15
SLC16
SLC22
SLC28
SLC29
SLCO
TAP
VDAC
